# Comparing the performance of OvaCyte and traditional techniques in detecting canine gastrointestinal parasites

**DOI:** 10.1186/s13071-025-06935-4

**Published:** 2025-08-13

**Authors:** Nagwa Elghryani, Conor G. McAloon, Geetika Lahan, Trish McOwan, Theo de Waal

**Affiliations:** 1 School of Veterinary Medicine, University College Dublin, Dublin, D04 D6F6 Ireland; 2 Telenostic Ltd, Kilkenny, R95 WN20 Ireland; 3 Department of Biology, Faculty of Arts and Sciences-Gamines, University of Benghazi, 33FX+QV9, Benghazi, Libya

**Keywords:** OvaCyte Telenostic, Canine gastrointestinal parasites, Ireland, OvaCyte, Canine, Gastrointestinal parasites, Traditional techniques

## Abstract

**Background:**

Companion animals are infected with a range of helminth and protozoan parasites which can have a significant effect on health and welfare. While several diagnostic techniques are available to detect parasitic infection, they all vary in sensitivity and specificity. This study aims to estimate the diagnostic performance of the OvaCyte™ Pet Analyser by comparing it with established benchmarks commonly used in reference laboratories.

**Methods:**

A total of 141 canine faecal samples, containing at least one species of parasite after screening using double centrifugation, were tested using four index tests: centrifugal flotation (faeces weighing either 1 g or 2 g), passive flotation, and the OvaCyte™ technique. The true status of each sample was determined on the basis of the initial screening test and the aggregated result of the four index tests. Sensitivity and specificity were calculated for each of the four index tests.

**Results:**

The OvaCyte™ Pet Analyser displayed high sensitivity ranging from 90% to 100% in detecting various parasite species in canines. Its sensitivity for roundworm and hookworm detection differed significantly from centrifugal flotation using 1 g and passive flotation techniques (*P* < 0.05). The OvaCyte™ demonstrated higher sensitivity in detecting *Cystoisospora* spp. (90%) and *Capillaria* spp. (100%) compared to all flotation methods (*P* < 0.001), though it showed slightly lower specificity than the other techniques.

**Conclusions:**

These results highlight the variability in sensitivity across different diagnostic methods, underscoring the importance of selecting the most reliable technique for accurate parasite detection in veterinary practice. However, the OvaCyte™ Pet Analyser exhibits an elevated level of sensitivity compared to other techniques.

**Graphical Abstract:**

**Figure:**
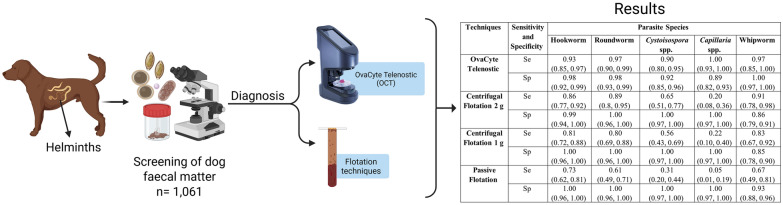

## Background

Gastrointestinal parasites (GIPs) pose major health problems in dogs [1–3]. These parasites, which include helminths such as *Toxocara* spp., *Toxascaris leonina*, *Uncinaria stenocephala*, *Ancylostoma* spp., *Trichuris vulpis*, *Capillaria* spp., and *Dipylidium caninum*, and protozoans such as *Giardia* spp., *Cystoisospora* spp., and *Sarcocystis* spp., cause a range of health issues in dogs, including diarrhoea, weight loss, anaemia, compromised immune response, and in severe cases, death [3–5].

In addition to causing clinical signs in dogs, several GIP species have the potential to cause zoonotic infection. Roundworms, particularly *Toxocara canis*, are responsible for toxocariasis in humans, a disease presenting in four clinical forms: visceral, ocular, covert, and neural [6]. This disease can have severe health repercussions, particularly in children [7]. While there have been occasional reports of human infection and disease associated with *T. leonina*, its zoonotic potential remains a subject of debate [8]. The zoonotic hookworms *U. stenocephala* and *Ancylostoma* spp. can cause a condition known as cutaneous larva migrans (CLM) [3]. The whipworm *T. vulpis* infects the large intestine of dogs and can cause diarrhoea, weight loss, anaemia, tenesmus, haemorrhagic colitis, or even death in dogs [3, 5].

*Cystoisospora* spp. are parasitic protozoa that can sometimes cause severe gastrointestinal distress, particularly when compounded by concurrent viral diseases or other immunosuppressive agents [9]. There are many species infecting dogs, but the clinically most important are *Cystoisospora canis* and *C. ohioensis* [9, 10]. These parasites invade the intestinal mucosal lining, resulting in signs such as watery diarrhoea, dehydration, anorexia, vomiting, and depression, and can be fatal in severe cases [9].

The prevalence of GIPs in dogs varies globally, ranging from 0.06% to 77%, influenced by region, climate, diagnostic methods, owners’ income levels in particular countries, and veterinary care [1, 4, 5, 11–13]. Preventive measures and prompt treatment are crucial for managing and mitigating the impact of GIP infections in dogs [9]. Therefore, regular examination of dogs for GIPs is critical for determining the extent of exposure and implementing appropriate control strategies in infected animals. Various diagnostic techniques are available for this purpose, each with distinct advantages and limitations [9, 14–16]. The direct faecal smear, though used for many years to detect GIPs, is often unreliable. Only a small sample of faeces is examined, which may not contain parasite eggs or oocysts if shedding occurs intermittently. Debris and artefacts in the sample can also make identification difficult, increasing the risk of false negatives or misinterpretations. Flotation methods are the most frequently employed for recovering parasite eggs and oocysts by leveraging differences in specific gravity (SG) between the egg(s), faecal debris, and flotation solution. Common flotation solutions include saturated sodium chloride (NaCl; SG 1.18), zinc sulfate (ZnSO_4_; SG 1.18–1.20), sugar (Sheather’s solution; SG 1.27–1.33), sodium nitrate (NaNO_3_; SG 1.20), and magnesium sulfate (MgSO_4_) [16]. Over the last century, there have been various semi-automated and automated techniques developed to accurately diagnose parasites, including FECPAK, FLOTAC, Mini-FLOTAC, Kubic FLOTAC, and automated robotic systems, serving both scientific research and commercial purposes [15]. The OvaCyte™ system by Telenostic Limited (OCT) is a sensitive diagnostic tool that is user-friendly, enabling rapid sample testing without the need for laboratory referral. Its technology facilitates image transmission for the future implementation of automated egg identification and counting. Recent studies have shown that OCT demonstrates equivalent efficacy in detecting and quantifying strongyle eggs in cattle and multiple GIPs in horses, compared to traditional techniques such as the McMaster and Mini-FLOTAC [17, 18]. However, to date, there have been no published studies on the accuracy of this technique for GIPs in dogs.

Therefore, the purpose of this study was as follows:

- Compare the performance of OCT with two commonly used flotation methods, passive flotation and centrifuge flotation, in recovering GIP eggs and oocysts from canine faecal samples
- Compare the performance of OCT and flotation techniques for detecting and counting canine *Cystoisospora* oocysts when the count is low
- Determine whether there is a significant difference in sensitivity (Se) and specificity (Sp) of the OCT between different scan types—standard, extended, and full scans—based on 100, 186, and 250 images captured, respectively

## Methods

### Sample collection

**Fig. 1:**
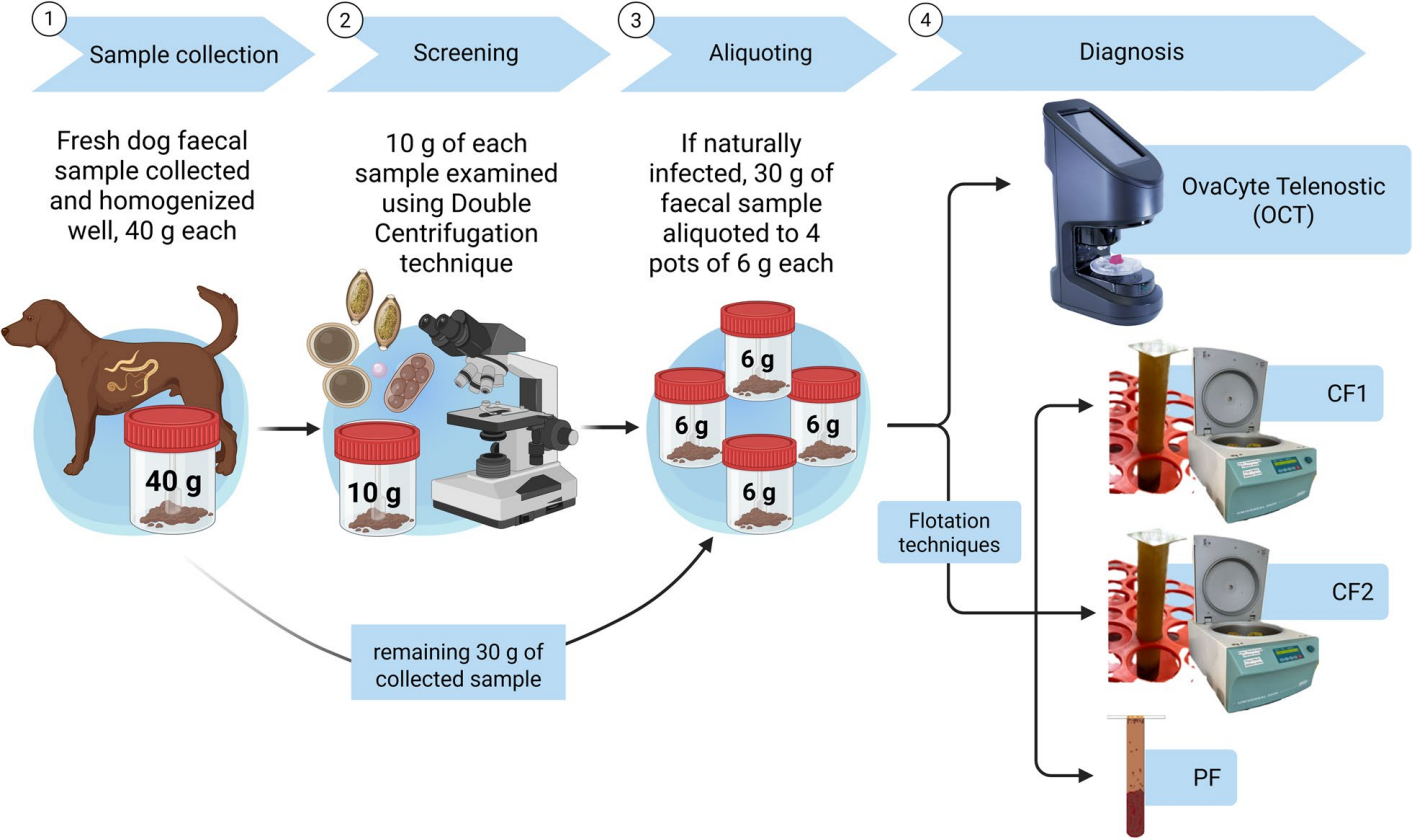
Study design and layout for the comparative analysis among four diagnostic techniques: OvaCyte Telenostic (OCT), centrifugal flotation 1 g (CF1) and centrifugal flotation 2 g (CF2), and passive flotation (PF)

A total of 1061 fresh faecal samples were collected, 40 g each from various locations across Ireland, including University College Dublin (UCD) Veterinary Hospital, veterinary practices, parks, shelters, and hunting dog kennels, between November 2023 and April 2024. Initially, the samples were screened for the presence of at least one parasite using a 10 g double-centrifugation technique. Of these, 143 samples tested positive for at least one parasite species. Two positive samples were discarded due to insufficient sample size (< 30 g) and the remaining 141 were further analysed using four index tests—(i) OCT, (ii) centrifugal flotation with 1 g of faeces (CF1), (iii) centrifugal flotation with 2 g of faeces (CF2), and (iv) passive flotation with 2 g of faeces (PF)—following standard protocols (see below). These tests were conducted within 24 h of sample collection. Additionally, 27 samples that were negative for all parasite species were included as negative controls. A schematic representation of the overall methodology used in this study is outlined in Fig. 1.

### Sample preparation and test procedure

#### Screening

Initially, all sourced samples were screened using the double-centrifugation technique with ZnSO_4_ (SG 1.2) [19]. In this technique, 10 g of each faecal sample was homogenised with 40 ml of water, filtered with a tea strainer, and centrifuged at 180×*g* for 5 min. The supernatant was discarded, and the deposit was mixed with flotation solution and centrifuged again at 180×*g* for 5 min. A coverslip was placed on the tube for 10 min, and the slides were examined under an Optika Microscope model B-800BF (Ponteranica, Italy) at 100× magnification.

#### Flotation Techniques using ZnSO_4_ (SG 1.2) [14]

After screening, the positive samples were examined by three flotation techniques: CF1, CF2, and PF. The faecal material was thoroughly mixed with 10 ml ZnSO_4_ (SG 1.2). For CF1 and CF2, the mixture was centrifuged at 180×*g* for 5 min in 15 ml test tubes. Following centrifugation, a coverslip was placed on top of the tube and left undisturbed for 5 min. In PF [14], the mixture was filtered using a tea strainer, and the filtrate was left to float in a 15 ml tube with a coverslip. Coverslips remained on the tubes for 5 min, and the slides were examined under a microscope (Optika Microscope model B-800BF, Ponteranica, Italy) at 100× and 400× magnification.

#### OvaCyte™ using Telenostic flotation fluid

**Fig. 2:**
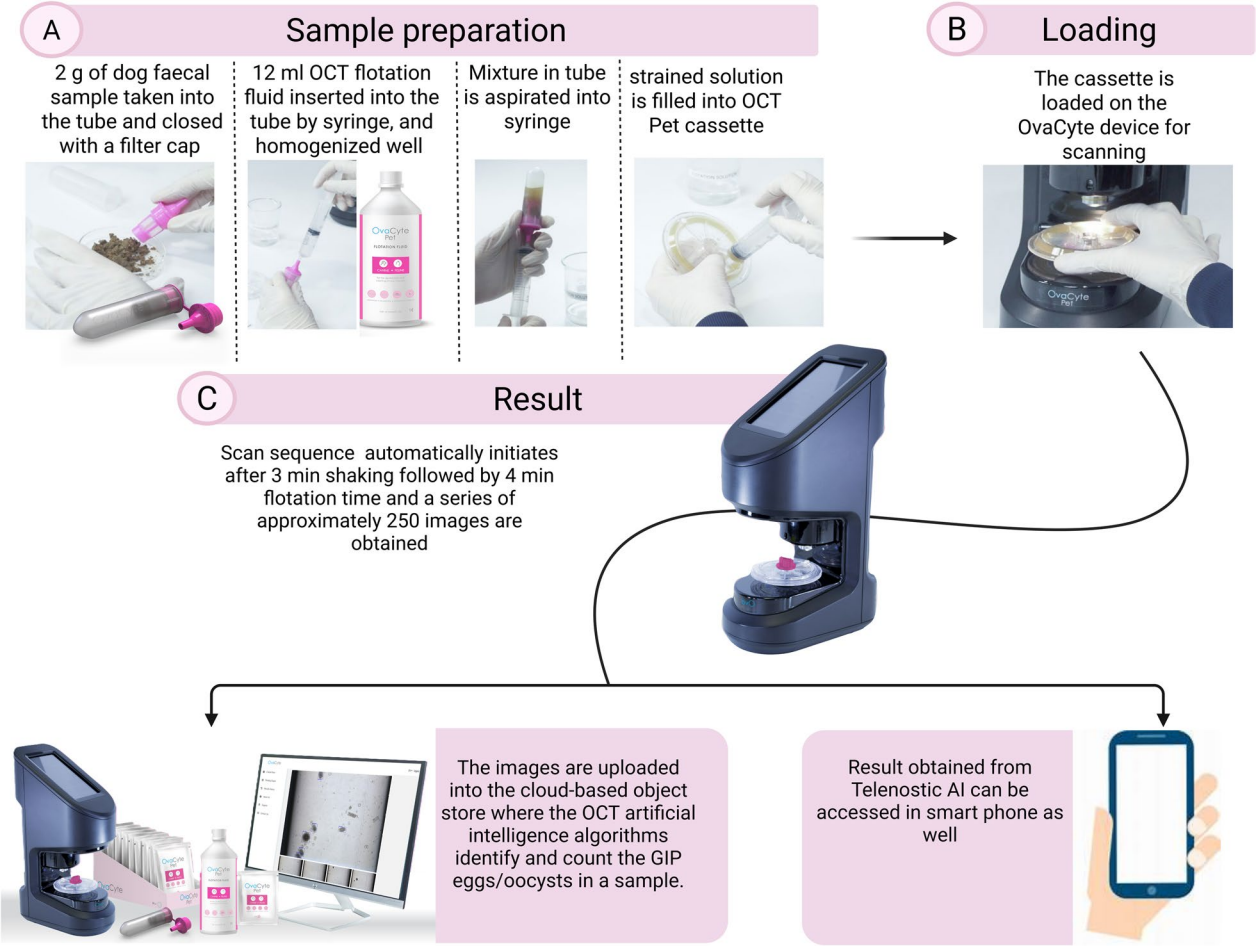
Overview of the OvaCyte Telenostic (OCT) technique for the diagnosis of GIPs (roundworm, hookworm, whipworm, and coccidia) in dog faeces

The OCT kit contains a plastic filter cap, a tube, and a new high-volume cassette. As illustrated in Fig. 2, 2 g of well-mixed faecal material was placed in the tube and sealed with a filter cap. A syringe was used to insert 12 ml of OCT flotation fluid into the tube. The mixture was then homogenised thoroughly by gently squeezing the tube. The homogenised slurry was drawn into a 20 ml syringe. Air was expelled from the solution in the syringe before transferring to the OCT Pet cassette, which was placed on the OCT instrument for scanning. After being started by the user, the automated sequence initiated the shaking phase followed by a flotation phase and then a series of image captures of approximately 250 images. The images were uploaded into the cloud-based object store, where the OCT artificial intelligence (AI) model identified and counted the GIP eggs/oocysts per gram (epg/opg) of each sample. Results were recorded as the number of eggs/oocysts that the model identified and counted, the number of images used by the model, the multiplication factor, and the resultant epg/opg for each sample/scan.

#### AI filter for distinguishing *T. vulpis* and *Capillaria* spp.

Further classification of the AI model’s identification of barrel-shaped eggs possessing a pair of polar plugs at each end was established based on the size of the major and minor axis of these egg types to determine whether they were either *T. vulpis* or *Capillaria* spp.

### Statistical analysis

The data were recorded in an Excel file (Microsoft Office 365, v2411), and the prevalence of each parasite was summarised as the percentage of positive samples, based on the screening technique results.

This study estimated the Se and Sp of the four index tests (i) OCT, (ii) CF1, (iii) CF2, and (iv) PF compared to the ‘true status’. Samples that tested positive for a particular parasite species using any of the index tests were considered the true status of infection with that parasite. Hence, true positive indicates the presence of that species using at least one of the index tests, and true negative indicates the absence of that species in all the index tests. Confidence intervals (CI, 95%) were calculated using the Clopper–Pearson (binomial) CIs.

To compare the Se and Sp between the standard, extended, and full scan, the results were individually evaluated for each set of images, the first 100 images, the first 186 images, and the full image set, and results were compared. To determine whether differences in Se and Sp between tests were significant, logistic regression models were developed on the subset of ‘true’ positive and ‘true’ negative samples, respectively, with each index test included as a predictor. Statistical analysis was conducted in R (R Core Team 2022) [20]. Binomial CIs were estimated using the ‘binom’ package [21], with statistical significance defined as *P* < 0.05.

In the case of *Capillaria*, complete separation occurred with all the positive samples returning a positive result for OCT. In this case, Firth’s bias reduction method was used [22] rather than standard logistic regression, using the ‘logistf’ package [23].

## Results

### GIP presence in the study sample and differences using the various methods

**Fig. 3:**
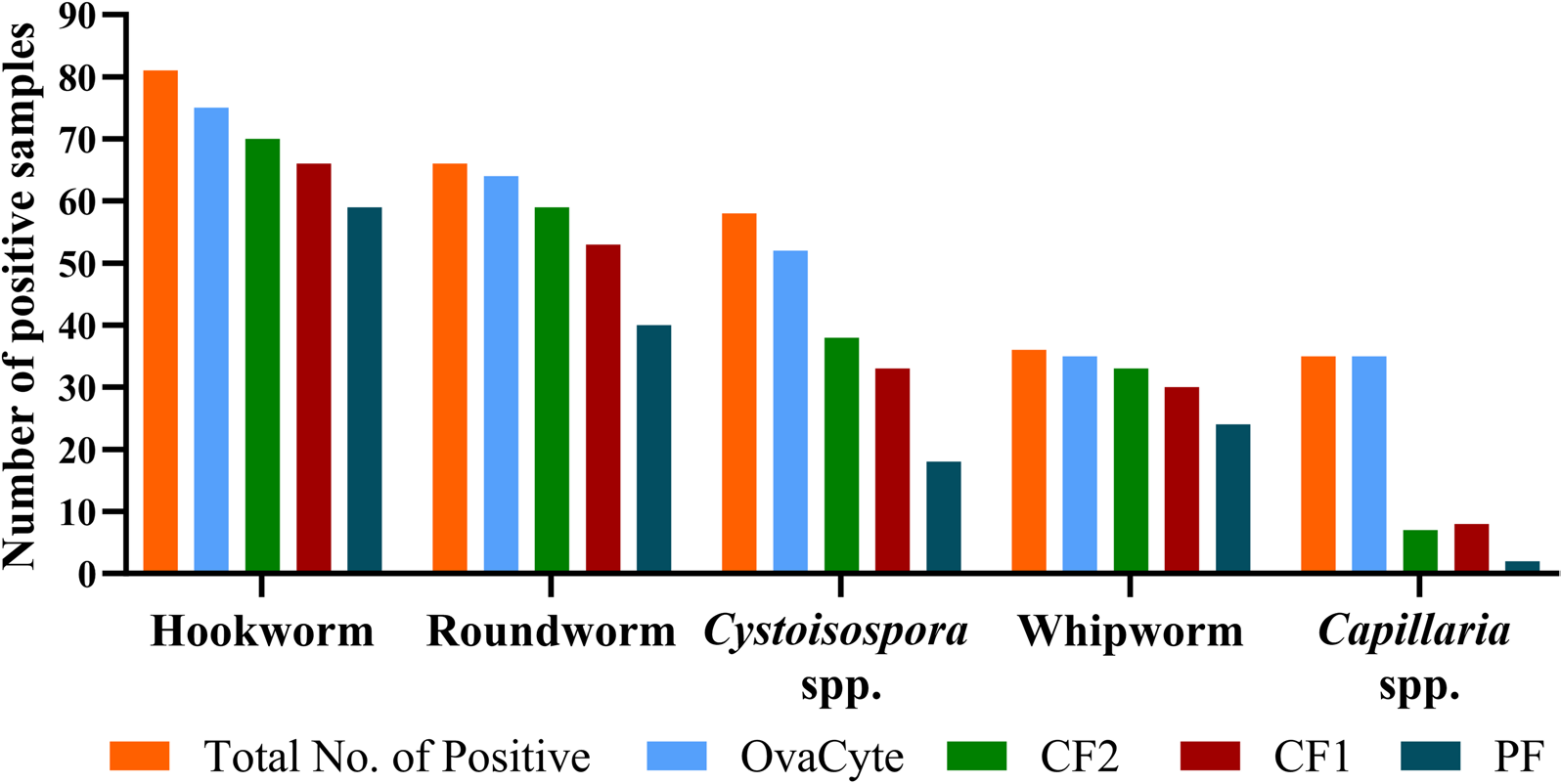
The total number of positive canine faecal samples (141) with gastrointestinal parasites detected using four different diagnostic techniques (OvaCyte, centrifugal flotation 1 g [CF1] and 2 g [CF2], and passive flotation [PF])

Of 1061 dog faecal samples screened using the double-centrifugation technique, the overall presence of infection with at least one GIP species was 13.5% (143/1061). Hookworm *U. stenocephala* was found as the most common parasite species, with 6.6% (70/1061), followed by roundworm at 5.4% (57/1061) and *Cystoisospora* spp. at 3.7% (39/1061). The OCT technique detected the highest number of positive samples for each parasite species, followed by the CF2 and CF1 techniques, respectively (Fig. 3). No significant difference was seen in the number of positive samples detected between OCT, CF1, and CF2 for hookworms, roundworms, or whipworms. However, for *Cystoisospora* spp. and *Capillaria* spp., OCT identified a significant number (*P* < 0.01) of positive samples in comparison to the CF1 and CF2 techniques. PF, on the other hand, detected the lowest number of positive samples for all parasitic species.

### Comparison of *Cystoisospora* spp. oocyst count levels using different techniques

The oocyst counts from the four index techniques, categorised according to oocyst count bands measured by the OCT technique, are shown in Table 1. Compared to the OCT technique, the Se of *Cystoisospora* counts at the lowest band (0–20) was relatively poor, especially for the PF technique (0.06). However, at the higher oocyst count bands, the Se of all the techniques increased (Table 1).

**Table 1:** The sensitivity of three different tests (passive flotation [PF], centrifugal flotation 1 g [CF1], centrifugal flotation 2 g [CF2]) for detecting *Cystoisospora* spp. in canine faecal samples, grouped by OvaCyte oocyst counts

OvaCyte count band	Technique	Sensitivity	No. of samples
(0, 20)	PF	0.06	35
	CF1	0.31	
	CF2	0.49	
(> 20, 100)	PF	0.33	15
	CF1	0.73	
	CF2	0.67	
> 100	PF	1.00	11
	CF1	1.00	
	CF2	1.00	

### Estimation of the sensitivity and specificity of the OvaCyte technique and the other three index techniques for each parasite

The Se and Sp of the four different index diagnostic techniques for detecting various parasites are presented in Table 2. For hookworm and roundworm, the OCT technique showed high Se (93% [0.85, 0.97]) and Sp (98% [0.92, 0.99]). Similarly, both CF1 and CF2 demonstrated high Se and Sp, while the PF technique was moderately sensitive in detecting both parasites, with perfect Sp (100%). For *Cystoisospora*, the OCT technique showed high Se (0.90 [0.80, 0.95]) and Sp (92% [0.85, 0.96]). The CF2 and CF1 techniques demonstrated low Se (65% [0.52, 0.78] and 56% [0.43, 0.69]) but perfect Sp (100%). The PF test, however, demonstrated low Se (31%) but still maintained perfect Sp (100%).

**Table 2:** The sensitivity (Se) and specificity (Sp) with 95% confidence intervals for the four techniques (OCT: OvaCyte Telenostic, CF2: centrifugal flotation 2 g, CF1: centrifugal flotation 1 g, PF: passive flotation) for gastrointestinal parasites in canine faecal samples

Techniques	Sensitivity and specificity	Parasite species				
		Hookworm	Roundworm	*Cystoisospora* spp.	*Capillaria* spp.	Whipworm
OCT	Se	0.93 (0.85, 0.97)	0.97 (0.90, 0.99)	0.90 (0.80, 0.95)	1.00 (0.93, 1.00)	0.97 (0.85, 1.00)
	Sp	0.98 (0.92, 0.99)	0.98 (0.93, 0.99)	0.92 (0.85, 0.96)	0.89 (0.82, 0.93)	1.00 (0.97, 1.00)
CF2	Se	0.86 (0.77, 0.92)	0.89 (0.8, 0.95)	0.65 (0.51, 0.77)	0.20 (0.08, 0.36)	0.91 (0.78, 0.98)
	Sp	0.99 (0.94, 1.00)	1.00 (0.96, 1.00)	1.00 (0.97, 1.00)	1.00 (0.97, 1.00)	0.86 (0.79, 0.91)
CF1	Se	0.81 (0.72, 0.88)	0.80 (0.69, 0.88)	0.56 (0.43, 0.69)	0.22 (0.10, 0.40)	0.83 (0.67, 0.92)
	Sp	1.00 (0.96, 1.00)	1.00 (0.96, 1.00)	1.00 (0.97, 1.00)	1.00 (0.97, 1.00)	0.85 (0.78, 0.90)
PF	Se	0.73 (0.62, 0.81)	0.61 (0.49, 0.71)	0.31 (0.20, 0.44)	0.05 (0.0, 0.19)	0.67 (0.49, 0.81)
	Sp	1.00 (0.96, 1.00)	1.00 (0.96, 1.00)	1.00 (0.97, 1.00)	1.00 (0.97, 1.00)	0.93 (0.88, 0.96)

In the case of *Capillaria* spp., the OCT technique achieved perfect Se of 100%; in contrast, the other techniques demonstrated low Se, indicating that many positive cases were missed, but still had perfect Sp (100%). For whipworm, both OCT and CF2 showed high Se at 100% and 91%, respectively, but moderate Sp. CF1 had moderate Se and Sp, at 83% and 85%, while PF demonstrated low Se and Sp (Table 2).

### Determining whether there is a significant difference between the sensitivity and specificity of the OCT compared to the other three techniques (CF2, CF1, and PF) for each parasite species

The significant difference in the Se of OCT compared to the other index techniques is shown in Table 3. The difference in Se of CF1 and PF, compared to OCT, for detecting roundworm and hookworm eggs was statistically significant (*P* < 0.05). Additionally, for detecting *Cystoisospora* (Table 3) and *Capillaria* spp. (Table 4), the Se of the OCT was significantly higher (*P* < 0.001) than that of all three flotation techniques. However, the difference in the Sp of the OCT compared to other index techniques was not significant.

**Table 3:** Significant differences between the sensitivity of the OCT (OvaCyte Telenostic) and the other three techniques (centrifugal flotation 1 g [CF1] and 2 g [CF2], and passive flotation [PF]) for gastrointestinal parasites in canine faecal samples

Parasite	Technique	Sensitivity			
		Estimate	Odds ratio	*P*-value	*Z*-value
Roundworm	OCT	Referent			
	CF1	−2.06	1.01	0.008	−2.64
	PF	−3.03	1.00	< 0.001	−3.99
Hookworm	OCT	Referent			
	CF1	−1.04	1.04	0.04	−2.04
	PF	−1.54	1.00	0.002	−3.13
*Cystoisospora*	OCT	Referent			
	CF2	−1.71	0.18	0.001	−3.40
	CF1	−2.05	0.13	< 0.001	−4.10
	PF	−3.09	0.05	< 0.001	−6.01

**Table 4:** Significant differences in sensitivity (Se) between the OCT (OvaCyte Telenostic) and the other three techniques (centrifugal flotation 2 g [CF2] and 1 g [CF1], and passive flotation [PF]) for *Capillaria* spp. detection in canine faecal samples

Technique	Coefficient	Se	Lower CI 0.95%	Upper CI 0.95%	Chi-square	*P*-value
Intercept	4.62	1.42	2.68	9.45	65.231	< 0.003
CF2	−6.37	1.48	−11.25	−4.25	∞	< 0.001
CF1	−6.22	1.47	−11.10	−4.12	∞	< 0.001
PF	−7.58	1.56	−12.53	−5.25	∞	< 0.001

### Differences in the sensitivity and specificity for OCT when estimating egg/oocyst counts using 100 images or 250 images compared to 186 images

The difference in the Se and Sp of the OCT technique when calculating epg/opg using either the standard or full scan compared to extended scan is summarised in Table 5. Although there is a slight decrease or increase for some GIPs in both Se and Sp when using 100 or 250 images compared to 186 images, these differences are not statistically significant (*P* < 0.05).

**Table 5:** Differences in the sensitivity and specificity for OCT (OvaCyte Telenostic) when estimating egg/oocyst counts using 100 images or 250 images compared to 186 images

Parasite	Images	Sensitivity			Specificity		
		Estimate	Odds ratio	*P*-value	Estimate	Odds ratio	*P*-value
	186	Referent			Referent		
Hookworm	100	−0.025	0.98	0.586	1.15E−02	1.01	0.583
	250	0.012	1.01	0.785	−5.26E−18	1.00	1.00
Roundworm	100	−0.076	0.93	0.154	9.80E−03	1.01	0.583
	250	0.076	1.08	0.154	5.83E−18	1.00	1.00
*Cystoisospora*	100	−0.098	0.91	0.142	0.028	1.03	0.30
	250	0.049	1.05	0.462	−0.009	0.99	0.729
*Capillaria*	100	−0.080	0.92	0.162	−8.32E−16	1.00	0.257
	250	0.100	1.11	0.081	−5.03E−29	1.00	1.00
Whipworm	100	−0.086	0.92	0.091	0.008	1.01	0.221
	250	0.029	1.03	0.571	0.008	1.01	0.221

## Discussion

The findings of this study, consistent with previous Irish research, indicate that hookworm and roundworm are the most common GIPs infecting dogs in Ireland [24]. In Europe, roundworm prevalence in dogs averages 10.8% [1], while hookworm prevalence ranges from 1.3% to 39.6%, influenced by factors such as region, climate, and veterinary resources. *Cystoisospora* spp. have been reported at prevalence rates ranging from 0.05% to 97.6% [25]. While various *Cystoisospora* spp. can infect dogs of any age, heavy infections are most common in puppies. Several studies indicate that the prevalence of patent *Cystoisospora* infections decreases with age. For instance, a German study by Barutzki et al. [10] found that dogs between 4 and 12 weeks old were more frequently infected than older dogs. Similarly, a study by Garcia et al. [11] in Ireland found that dogs younger than 3 months were more likely to be infected.

*Capillaria* spp. and whipworm (*T. vulpis*) are relatively rare across Europe, ranging from 0.4% to 0.8% and 0.3% to 9.9%, respectively [3–5, 12, 26, 27]. However, a study in 2023 in Italy recorded prevalence of 19.51% (95% CI 10.23–34.01) for *Capillaria* spp. (*Capillaria aerophila*, syn. *Eucoleus aerophilus*) [28]. The study also identified the red fox as the primary reservoir and transmitter of * C. aerophila * to both wild and domestic animals, including dogs and cats. Despite its significance as a lung parasite in companion animals, *Capillaria* spp. remain under-recognised by veterinarians, likely due to the limited parasitological research in this field and low levels in faeces. Diagnosing * C.* *aerophila* through coprological techniques is challenging because its eggs closely resemble those of the whipworm *T. vulpis* [29]. 

Accurate diagnostic testing is essential for the detection and management of parasitic infections. Sensitivity and sp are critical in evaluating diagnostic tests, particularly for screening parasitic infections, where false negatives can lead to untreated infections, and false positives might cause unnecessary treatments. The data from this study provided a comparison of four different diagnostic techniques (OCT, CF2, CF1, and PF) for detecting GIPs in canines, including their Se and Sp. There was a notable difference in Se among the four techniques in all parasite species, with OCT the most sensitive and PF the least sensitive for all parasites.

In this study, the Sp of most of the index techniques remains consistently high, ranging from 83% to 100%. This suggests that the index techniques, with their high specificity, are effective at ruling out non-infected individuals. Since there are few or no false positives, those who test positive are true positives, minimising overtreatment.

The diagnostic accuracy of different techniques used in this study varied significantly depending on the specific parasite (Table 2). While many techniques exhibited high Sp, their Se tended to be more variable. For hookworms, roundworms, and whipworm, the OCT and flotation centrifuge techniques (CF2) demonstrate perfect Se and Sp, making them highly effective for detecting eggs of these helminths. However, both CF1 and PF show a statistically significant reduction (*P* < 0.05) in Se compared to OCT, particularly when parasite egg counts were low, making them less reliable for detecting hookworms and roundworms (Table 3). This reduced Se is crucial, as it could lead to misdiagnosis, leaving infected individuals untreated. Additionally, for *Cystoisospora* spp., the flotation techniques (CF2, CF1, PF) demonstrate moderate to poor Se around 30–60% at low oocyst counts, suggesting they miss a substantial number of true positives. However, their high Sp of 100% (0.97, 1.00) makes them reliable for confirming positive results, though they remain unreliable for ruling out infection due to the risk of false negatives. In contrast, OCT demonstrated higher Se and was able to detect more positive samples, especially those with very low oocyst counts (fewer than 20 oocysts) (Table 2). However, OCT requires further development, as its Sp is less than that of flotation techniques, often falsely identifying negative samples as positive due to debris interference (Table 3).

For *Capillaria* spp., both flotation techniques CF2 and CF1 perform poorly, with Se of only 16% and 14%, respectively. This indicates that these methods only correctly identify about one-quarter of true positives. Overall, the OCT detected more *Capillaria* spp.-positive samples than the others. It is possible that the diagnostic techniques employed in other surveys [4, 5, 24, 26, 30] underestimated the true prevalence of *Capillaria* spp. Recent studies highlight the limitation of flotation techniques in detecting *Capillaria* eggs, which can lead to missed or underdiagnosed cases in dogs [28, 29]. *Capillaria* eggs are often misdiagnosed as *T. vulpis* due to their similarities in shape and size, affecting treatment efficacy and epidemiological assessment [28, 29, 31]. The use of AI models has improved the detection of mixed-species infections by identifying parasites beyond what traditional methods capture [15, 32]. This approach addresses the limitations of microscopy, which requires expert skills to distinguish *Capillaria* spp. eggs from those of morphologically similar species. Furthermore, relying solely on manual slide reading introduces risk, as it often lacks consensus and depends heavily on individual interpretation [15, 28]. Considering all the above, several technical factors contribute to variations in faecal flotation results, including the volume and weight of the faecal samples, the processes of filtration and homogenisation, the use of centrifugation, and the selection of flotation solutions [14–16, 32]. A key finding of this study is that reducing the weight of faecal samples to 1 g in CF significantly decreased Se, making it unsuitable for low egg counts. The PF technique, which uses a simpler process without centrifugation, was once commonly used for GIP detection. However, it has since been shown to have low Se, accuracy, and precision, primarily due to the absence of centrifugation as well as the short flotation time, which falls well below the standard recommended flotation time of at least 10 min [15, 31]. This technique is now regarded as crude and prone to false negatives, allowing only qualitative rather than quantitative diagnoses [32].

Among the faecal flotation techniques, centrifuged flotation has been considered a standardised method in both laboratory and field settings for many decades. However, like other flotation techniques such as Ovaassay and simple flotation [14–16], it requires specialised personnel and is time-intensive, especially when processing large numbers of samples, as in epidemiological surveys. Extended slide reading times can result in missed positive samples, especially when some parasites are present in high numbers while others appear in lower numbers, increasing the likelihood of overlooking those in low abundance. This risk is also present when eggs or oocysts are abundant but accompanied by multiple species, or when slides are obscured by debris; certain species or low-prevalence organisms can go undetected [33, 34]. Taken together, sample processing and identification are crucial steps that, if done incorrectly, can affect the diagnostic value of the technique. However, the OCT system offers accurate detection and quantification of GIPs in dog faeces, particularly in settings where the centrifugation step cannot be performed, such as in veterinary practices. It is user-friendly and can be operated by anyone by following the manufacturer’s instructions, without the need for specialised training. Additionally, the system allows images to be transmitted via the internet to other laboratories, enabling remote use from different locations or countries. This makes the OCT system a useful tool for establishing a network of laboratories or supporting field operators directly. The OCT device enhances operator safety compared to other copromicroscopic techniques, as it functions as a closed system. The technician only encounters the sample during the homogenisation process, before filling the OCT.

This study has several limitations that may impact the generalisability of its findings. First, the small sample size for whipworm and *Capillaria* spp. detection limits the statistical power needed to draw definitive conclusions on the effectiveness of different flotation techniques across diverse populations. Factors such as slide contamination from debris could also have contributed to missed positives or false negatives.

One limitation of the OCT test is its cost. In regions where labour costs are low and testing volumes are high, a human operator may be more cost-effective than the automated system. However, consideration must be given to availability and training of the operator, and to traceability and repeatability of analysis to accurately reflect the true cost of the test. Another limitation concerns plastic usage. Currently, OCT cassettes are single-use, as basic cleaning between uses risks cross-contamination, which could lead to inaccurate results and unnecessary anthelmintic treatments. This not only affects test accuracy but also contributes to excessive plastic waste. To address this, Telenostic is introducing a dual-cassette design that will effectively halve the plastic used per test. This design not only minimises plastic waste but also lowers the overall cost and time per test, contributing to greater operational efficiency. In addition, a dedicated wash station is under development to thoroughly flush and sterilise cassettes, enabling safe reuse. This will help minimise both plastic waste and the risk of cross-contamination. The dual-cassette system and the wash station offer the potential to reduce both the cost and environmental impact of the test.

## Conclusions

In conclusion, these findings underscore the variability in Se across diagnostic methods, emphasising the need for reliable approaches to ensure accurate parasite detection in veterinary practice. The OvaCyte™ Pet Analyser demonstrates exceptional Se and Sp in detecting GIPs in dogs, excelling particularly in the identification of *Cystoisospora* spp. and *Capillaria* spp. Its ability to distinguish *Capillaria* based on size with 100% Se and 89% Sp within our sample highlights its potential as an advanced diagnostic tool compared to traditional flotation techniques. These results reaffirm the importance of integrating advanced diagnostic technologies to enhance parasite detection and elevate the standard of veterinary care.

## Data Availability

Data supporting the main conclusions of this study are included in the manuscript.

## Abbreviations

GIP: Gastrointestinal parasite
OCT: OvaCyte Telenostic
CF1: Centrifugal flotation with 1 g of faeces
CF2: Centrifugal flotation with 2 g of faeces
PF: Passive flotation
